# The Role and Therapeutic Potential of the STING Signaling Pathway in the Pathogenesis of Diabetic Nephropathy

**DOI:** 10.3390/ph19060927

**Published:** 2026-06-12

**Authors:** Xin-Yuan Zhang, Yan Hu, Ming-Tan Tang

**Affiliations:** 1 School of Basic Medical Sciences, Shandong University, Jinan 250012, China; 202400411073@mail.sdu.edu.cn (X.-Y.Z.); 202416399@mail.sdu.edu.cn (Y.H.); 2 School of Pharmaceutical Sciences, Shandong University, Jinan 250012, China; 3 State Key Laboratory of Discovery and Utilization of Functional Components in Traditional Chinese Medicine, Shandong University, Jinan 250012, China; 4 National Medical Products Administration Key Laboratory for Technology Research and Evaluation of Drug Products, Shandong University, Jinan 250012, China; 5 Shandong Key Laboratory of Targeted Drug Delivery and Advanced Pharmaceutics, Shandong University, Jinan 250012, China

**Keywords:** cGAS-STING, diabetic nephropathy, inflammatory response, apoptosis, fibrosis, STING inhibitor

## Abstract

Diabetes mellitus currently represents a major public health burden worldwide. Among diabetic individuals, diabetic nephropathy (DN) is a frequent and serious microvascular complication that markedly affects both patients’ quality of life and clinical outcomes. DN has also emerged as the leading contributor to end-stage renal disease (ESRD). Over recent years, the stimulator of interferon genes (STING) signaling pathway (an essential element of the innate immune system) has drawn substantial research interest because of its involvement in inflammation and cell injury. This article reviews the fundamental mechanisms of the STING pathway and its regulatory functions in the pathogenesis of DN, with a focus on how the STING pathway mediates inflammatory responses, apoptosis, and fibrosis in diabetic renal tissues. Additionally, combining the latest findings from preclinical and clinical research, we discuss potential therapeutic strategies targeting the STING pathway. Beyond traditional STING inhibitor therapies, we highlight the emerging field of precision medicine for DN, summarizing recent research achievements in gene intervention, such as CRISPR-based gene editing, RNA interference (RNAi) technologies, and combination therapy strategies. Distinct from prior reviews, this work discusses the emerging concept that STING may function as a molecular hub connecting inflammation, fibrosis, and cell death in DN, while emphasizing that this concept is mainly supported by preclinical and early human observational evidence. Through this comprehensive review, we aim to enhance our understanding of the role of the STING signaling pathway in DN, identify novel therapeutic targets, and provide theoretical perspectives for the prevention and treatment strategies that require further clinical validation.

## 1. Introduction

Diabetes has become a global public health crisis. In 2024, approximately 589 million adults worldwide were living with diabetes, representing 11.1% of the adult population, with more than 40% remaining undiagnosed. The number of affected individuals is projected to rise to 853 million by 2050. Type 2 diabetes accounts for more than 90% of all cases [1]. Diabetes is responsible for more than 3.4 million deaths annually, emphasizing its substantial contribution to premature mortality and global health loss. With the growing epidemic of diabetes, the burden of diabetic nephropathy (DN) has increased substantially. Globally, approximately 30–40% of patients with diabetes develop DN. More specifically, a Global Burden of Disease 2021-based analysis estimated 107.6 million prevalent cases of DN worldwide in 2021. In the same year, DN accounted for approximately 477.3 thousand deaths, with an age-standardized mortality rate of 5.7 per 100,000 population, representing a 37.8% increase since 1990. These data indicate that DN is not only a major cause of ESRD but also an increasingly important contributor to diabetes-related mortality [2,3].

The progression of DN to ESRD typically takes 10–20 years, though marked inter-individual variability exists. Notably, diabetic nephropathy is associated with the most rapid decline in eGFR compared to other etiologies. Patients with biopsy-proven DN have a significantly higher risk of progressing to ESRD than those without DN (hazard ratio 2.56) [4]. Mechanistically, DN progression involves hyperglycemia-induced mitochondrial dysfunction and oxidative stress, which activate inflammatory cascades, including the IL-17, Wnt, and RAGE signaling pathways. Immune infiltration, particularly of M1 and M2 macrophages, plays a critical role in disease progression [5]. Key risk factors accelerating DN progression include proteinuria, hypertension, older age, and cardiovascular comorbidities [5]. Traditional indicators such as albuminuria frequently identify renal impairment only at advanced stages, limiting early intervention [6].

Nevertheless, despite strict glycemic and blood pressure control, a considerable proportion of patients continue to progress to renal failure, suggesting that current therapeutic strategies inadequately target the core pathological mechanisms of the disease [7]. Current therapies, such as RAAS inhibitors and SGLT2 inhibitors, primarily target hemodynamic and metabolic pathways, leaving persistent inflammation, fibrosis, and metabolic memory largely unaddressed—evidenced by the fact that even in landmark trials like CREDENCE, an 11% residual risk of kidney failure remained after just 2.6 years of combination therapy [8]. Additionally, disease heterogeneity, such as the non-albuminuric DN phenotype (accounting for 30–40% of cases), limits the effectiveness of one-size-fits-all approaches [9]. The marked inter-individual variability in the rate of renal function decline also suggests that susceptibility factors—including genetic background and chronic inflammation—play important modifying roles in disease progression [10].

Hyperglycemia-induced metabolic disturbances trigger a cascade of cellular and molecular reactions, ultimately leading to structural and functional impairments such as glomerulosclerosis, tubulointerstitial fibrosis, and progressive renal function loss. The pathogenesis is complex and involves multiple factors, including metabolic dysregulation, oxidative stress, persistent inflammatory responses, and programmed cell death, among other pathological processes. The complex interactions among these pathological mechanisms not only highlight the challenges in elucidating the pathophysiological mechanisms of DN, but also underscore the necessity of identifying novel molecular targets to improve therapeutic outcomes [11]. In recent years, the role of innate immune systems and inflammatory signaling pathways in DN has garnered increasing attention, as chronic inflammation has been established as a key driver of DN and fibrosis progression [12].

Among the emerging molecular mechanisms implicated in DN, the STING signaling pathway—known for its essential function in innate immune surveillance and inflammatory control—has attracted considerable research interest [13]. STING is an adaptor protein on the endoplasmic reticulum membrane that is activated upon recognition of cytosolic double-stranded DNA (dsDNA) by its upstream sensor cGAS, which generates the second messenger 2′3′-cGAMP. This activates STING, which then recruits TBK1, ultimately driving the transcription of type I interferons and proinflammatory cytokines via IRF3 and NF-κB [14,15]. Conversely, under metabolic stress or mitochondrial injury, endogenous dsDNA leaks into the cytoplasm, causing aberrant cGAS-STING activation and promoting a chronic inflammatory microenvironment [16,17].

Within the context of DN, various stressors such as hyperglycemia, lipotoxicity, and hemodynamic disturbances contribute to mitochondrial dysfunction in renal tubular epithelial cells. This dysfunction triggers the release of mitochondrial DNA (mtDNA) into the cytoplasm, which in turn leads to aberrant activation of the cGAS-STING signaling axis [18]. Animal model studies have confirmed that STING is significantly upregulated in DN kidneys, and its activation is closely associated with tubulointerstitial inflammatory infiltration, myofibroblast activation, and collagen deposition. Pharmacological or genetic inhibition of STING can significantly alleviate proteinuria, renal fibrosis, and deterioration of renal function in these preclinical models [18]. These findings support the pathogenic role of the STING pathway in the progression of DN and suggest its potential as a molecular link between metabolic disorders and chronic inflammation, although its relative contribution compared with other inflammatory pathways in human DN remains incompletely defined [19]. This review aims to systematically elucidate the biological functions of the STING signaling pathway, provide an in-depth analysis of STING-mediated inflammation and fibrosis mechanisms in DN, evaluate therapeutic strategies targeting this pathway, and explore its clinical translation prospects and challenges.

It should be noted, however, that this proposed hub-like role of STING remains primarily supported by preclinical animal models and in vitro studies. Direct evidence from human DN samples is still limited, and the relative contribution of STING activation compared to other inflammatory pathways, such as NF-κB, in human disease progression has not been fully elucidated. Furthermore, the spatiotemporal dynamics of STING activation across different cell types and disease stages in DN require further investigation.

## 2. STING Signaling Pathway

### 2.1. STING Structure and Canonical Activation

STING is a core component of the innate immune system, primarily localized on the ER membrane. Structurally, STING is a transmembrane protein anchored in the ER by four transmembrane helices, followed by a cytoplasmic ligand-binding domain (LBD) responsible for sensing cyclic dinucleotides (CDNs) such as cyclic guanosine monophosphate- adenosine monophosphate (cGAMP) [20,21]. LBD forms a dimeric structure, and conformational changes occur upon ligand binding, thereby activating STING [22]. In the context of DN, sustained hyperglycemia-induced mitochondrial damage leads to persistent mtDNA leakage and subsequent cGAMP production, which may continuously drive this activation process [18,23]. This conformational rearrangement leads to the release of the C-terminal tail (CTT), enabling STING to oligomerize and form higher-order structures, thereby providing a foundation for downstream signal transduction [24,25]. Of note, enhanced STING signaling has been observed in diabetic kidney tissues, correlating with the severity of renal injury [26,27].

**Figure 1:**
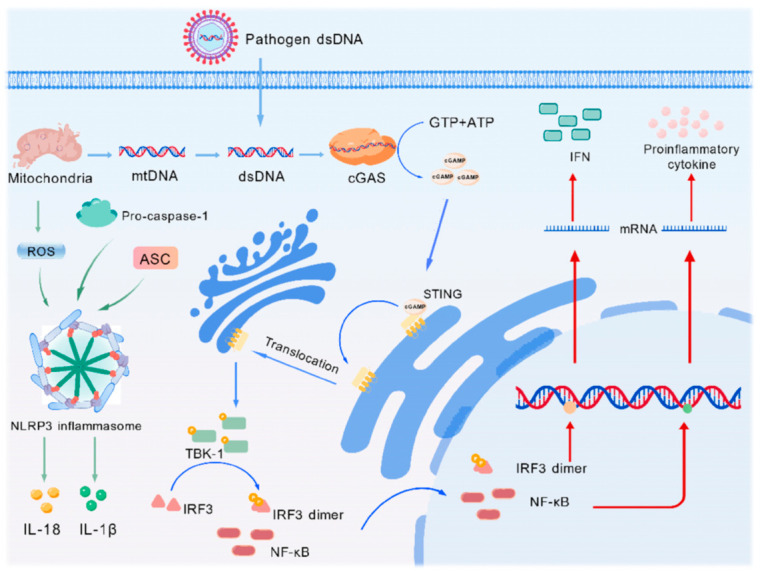
Structure and activation mechanism of STING. STING functions as a key adaptor protein residing on the endoplasmic reticulum (ER) membrane. The activation cascade of this molecule is triggered upon cytosolic detection of double-stranded DNA (dsDNA) by cGAS, which subsequently generates 2′3′-cGAMP—a ligand that binds to STING with strong affinity. Following this ligand engagement, STING experiences conformational rearrangements, undergoes oligomerization, and migrates from the ER toward the Golgi apparatus. Throughout this intracellular trafficking process, STING engages and activates TBK1, which then phosphorylates IRF3. Once phosphorylated, IRF3 forms homodimers and relocates to the nucleus, where it drives the transcription of genes encoding type I interferons (IFNs) and various proinflammatory cytokines. Beyond this canonical route, STING signaling also triggers the NOD-like receptor family pyrin domain-containing 3 (NLRP3) inflammasome cascade, thereby promoting the maturation and subsequent release of interleukin (IL)-1β and IL-18, which further intensify the inflammatory state. Full terms for all abbreviations can be found in the Abbreviations Section.

The initiation of STING activation occurs upon cytoplasmic recognition of dsDNA by cGAS. This enzyme subsequently drives the production of 2′3′-cGAMP, a second messenger that binds with high affinity to STING localized on the endoplasmic reticulum [28,29,30]. Once cGAMP engages the ligand-binding domain (LBD) of STING, it triggers conformational rearrangements and promotes the translocation of this protein from the ER to the Golgi apparatus [20,31]. During this trafficking process, STING recruits and activates TBK1, which in turn phosphorylates IRF3, thereby inducing IRF3 dimerization. This event facilitates the nuclear entry of IRF3 and ultimately drives the transcription of genes encoding type I interferons (IFNs) as well as various pro-inflammatory cytokines [29,32]. Collectively, this signaling cascade represents the canonical STING pathway, which serves an essential function in antiviral defense and immune surveillance. The Canonical activation pathway of STING is illustrated in Figure 1.

Furthermore, the STING activation pathway also involves the recruitment of other signaling molecules, which synergize with IRF3 to amplify the inflammatory response [33]. This pathway also integrates multiple signaling sources from cytoplasmic DNA, including mtDNA released during cellular stress or injury, which can activate the cGAS-STING signaling pathway and lead to aseptic inflammation [34,35]. Notably, bacterial infections can utilize the STING pathway by delivering bacterial DNA to host cells, thereby activating STING and modulating the host immune response [36].

In summary, STING serves as a pivotal signal sensor and transducer in the innate immune system, leveraging its dual functions of ER membrane localization and ligand-binding domain. When cytoplasmic dsDNA is detected by the cGAS sensor and cGAMP is generated, STING undergoes oligomerization activation, followed by translocation to recruit TBK1 and IRF3 proteins. This process ultimately triggers type I IFN production and the expression of inflammation-related genes. This precisely regulated molecular mechanism forms the foundation of the immune response in combating infections and cellular damage.

### 2.2. Regulatory Mechanisms of the STING Signaling Pathway

#### 2.2.1. Post-Translational Modifications

The STING signaling pathway is governed by complex regulatory mechanisms to maintain immune homeostasis and prevent excessive inflammation. Negative regulation is primarily achieved through post-translational modifications, such as controlled protein degradation, ubiquitination, and phosphorylation. These mechanisms collectively prevent the overactivation of STING and its downstream signaling pathways [31,37,38]. Similarly, the catalytic subunit of protein phosphatase 6 (PPP6C) negatively regulates the activation of STING by dephosphorylating it, thereby limiting the sustained type I IFN response that may trigger autoimmune responses [39]. Additionally, E3 ligases such as tripartite motif containing 41 (TRIM41) ubiquitinate STING, marking it as a proteasome degradation target, thereby regulating the intensity and duration of the immune response [40]. Furthermore, the palmitoylation modification of STING at specific cysteine residues is critical for its activation and transport. Enzymes involved in fatty acid synthesis, such as fatty acid synthetase (FASN), regulate this modification process to link metabolic status with STING activity [41]. As a precursor of fatty acid synthesis, malonyl-CoA can inhibit the palmitoylation modification and activation of STING, revealing a metabolic checkpoint in STING regulation [41]. While direct evidence linking hyperglycemia to STING palmitoylation or ubiquitination in DN is currently lacking, inhibitors targeting these modifications, such as C-176 and H-151, have been reported to suppress STING activation [42,43], and their potential efficacy in DN warrants future investigation.

#### 2.2.2. Metabolic and Microenvironmental Regulation

The cell environment also affects the activity of STING. Mitochondrial dysfunction can lead to mtDNA leakage into the cytoplasm, activating the cGAS-STING signaling pathway and triggering inflammatory responses [34,44,45]. Isopentenyl diphosphate isomerase 1 (IDI1) can inhibit this signaling pathway by promoting the degradation of cGAS protein, thereby linking metabolic reprogramming with the innate immune system [40]. Furthermore, hypoxia and endoplasmic reticulum stress (ERS) can modulate STING signaling, integrating cellular stress responses with immune activation mechanisms [33,46,47]. In the context of DN, mitochondrial dysfunction-induced mtDNA leakage is a well-established upstream event driving STING activation [18,23].

#### 2.2.3. Phase Separation and Other Emerging Regulatory Mechanisms

Recent studies have revealed that protein phase separation serves as an important regulatory mechanism in the STING signaling pathway. Upon activation, STING undergoes phase separation to form dynamic condensates, which function as signaling platforms that recruit and enrich downstream kinases such as TBK1, thereby promoting the efficient assembly and propagation of signaling complexes. Beyond signal amplification, phase separation also contributes to the fine-tuning of STING activity. For instance, excessive cGAMP induces STING to form unique puzzle-like condensates that sequester surplus cGAMP and TBK1, preventing hyperactivation and maintaining immune homeostasis [48]. Furthermore, extracellular vesicles (EVs) can deliver STING oligomers or mitochondrial DNA to neighboring cells, modulating immune responses through paracrine mechanisms [49,50]. Studies have shown that Evs released from stressed cells can carry STING activators, thereby triggering cGAS-STING signaling in recipient cells and enabling intercellular signal propagation [49]. In the DN kidney, Evs released from damaged tubular epithelial cells and podocytes may mediate the spread of STING signals within glomerular and interstitial compartments, transmitting local mitochondrial damage signals to surrounding cells and amplifying inflammatory responses. However, this hypothesis remains to be experimentally validated, and the role of Evs in DN-associated STING activation is currently an open area of investigation.

These regulatory mechanisms function collectively to maintain appropriate activation levels of the STING pathway. This balanced activation serves a dual purpose: it curbs excessive inflammatory reactions and autoimmune responses while still effectively promoting protective immunity. In DN, a deeper understanding of the above regulatory mechanisms may facilitate the development of STING-targeted therapeutic strategies and open new avenues for precision medicine in DN.

### 2.3. Crosstalk Between STING and Other Inflammatory Signaling Pathways

The STING signaling pathway does not function in isolation, but rather forms a complex interactive network with key inflammatory pathways such as the NF-κB pathway, amplifying and modulating immune responses through synergistic effects. Based on current evidence, these interactions can be categorized into direct molecular interactions and downstream convergence.

#### 2.3.1. Direct Crosstalk with the NF-κB Pathway

The STING pathway directly interacts with the NF-κB pathway. STING activation triggers two distinct signaling branches in a temporally and spatially regulated manner. At the Golgi apparatus, STING recruits TBK1 and phosphorylates IRF3. Through a delayed mechanism involving monomeric IRF3 as an adaptor, STING further recruits the TRAF6-LUBAC complex, which synthesizes M1-linked ubiquitin chains, thereby activating the classical NF-κB pathway and leading to the transcription of pro-inflammatory cytokines such as IL-15, TNF-α, and IL-6. Of note, the NF-κB response is enabled only within a short time window following STING exit from the Golgi, prior to its lysosomal degradation [32,33,51,52].

#### 2.3.2. Indirect Crosstalk with Inflammasome and Cell Death Pathways

The STING signaling pathway exhibits crosstalk with the NLRP3 inflammasome. The NLRP3 inflammasome is a multi-protein complex that governs the processing and release of IL-1β and IL-18, two critical regulators of inflammatory reactions and pyroptosis [53]. Through its detection of cytoplasmic DNA, the cGAS-STING axis induces mitochondrial stress and generates reactive oxygen species (ROS), which in turn facilitates the recruitment and activation of the NLRP3 inflammasome [44,53]. This reciprocal interaction establishes a positive feedback loop that sustains and amplifies the inflammatory response, thereby contributing to the development of multiple inflammatory and autoimmune disorders.

Meanwhile, the STING signaling pathway also extends to other cell death pathways such as apoptosis, necroptosis, and ferroptosis. In these processes, STING activation directly influences cell fate determination and inflammatory responses [54,55]. For instance, STING recruits TBK1 and phosphorylates IRF3, which not only activates the NF-κB pathway but also induces type I interferon production, and the latter can regulate the expression of ferroptosis-related genes. In addition, necroptosis signals promote mitochondrial DNA release, thereby activating the cGAS-STING complex [56]. This signaling pathway integration mechanism not only highlights the complexity of innate immune regulation, but also reveals the pivotal role of STING as a central hub coordinating inflammation and cell death.

In conclusion, the STING pathway exhibits extensive interactions with the NF-κB and other signaling pathways, collectively regulating comprehensive inflammatory responses. In DN, the above crosstalk mechanisms have been linked to specific pathological events: the STING-NF-κB axis promotes the transcription of TNF-α, IL-6, and MCP-1, recruiting macrophages and exacerbating glomerular inflammation [26,27]; the STING-NLRP3 axis triggers Cysteine-aspartic acid protease 1 (caspase-1) activation and GSDMD-mediated pyroptosis in renal tubular epithelial cells and podocytes, leading to albuminuria and nephron loss [57]. These findings suggest that targeting STING or its interacting pathways may offer therapeutic opportunities for DN, such as inhibiting STING palmitoylation with C-176/H-151 to block NF-κB-driven inflammation, suppressing NLRP3 activation to attenuate pyroptosis, or modulating cell death pathways to preserve renal function. A detailed discussion is provided in the following sections.

## 3. STING Signaling Pathway Activation and Pathogenic Effects in DN

The typical clinical manifestations of DN include proteinuria and progressive renal failure. These conditions are not solely caused by hemodynamic changes but rather represent a complex pathological process driven by three core events: chronic inflammation, renal fibrosis, and death of renal tubular epithelial cells [3]. Hyperglycemia-induced metabolic stress activates multiple inflammatory pathways, promoting macrophage infiltration and the release of factors such as IL-1β, TNF-α, and transforming growth factor-β (TGF-β), which disrupt the structure of podocytes and compromise the integrity of the glomerular filtration barrier, leading to the development of proteinuria [18]. Chronic inflammation subsequently stimulates fibroblast activation and drives the mesenchymal transition of renal tubular epithelial cells. These cellular events result in widespread extracellular matrix (ECM) accumulation, which in turn promotes glomerulosclerosis and tubulointerstitial fibrosis. Consequently, the population of functional nephrons progressively declines [58,59]. Meanwhile, oxidative stress and mitochondrial dysfunction trigger apoptosis and programmed necrosis of renal tubular epithelial cells, accelerating the loss of renal parenchyma [60,61]. These three factors mutually reinforce each other, forming a vicious cycle of “inflammation–fibrosis–cell death”, ultimately leading to a decline in glomerular filtration rate (GFR), which manifests as clinical renal failure. Although glycemic control and blood pressure management are fundamental therapeutic strategies, a significant number of patients continue to experience disease progression, suggesting that deeper pathological mechanisms remain incompletely elucidated [62]. Among them, the cGAS-STING pathway, as a key bridge linking metabolic disorders and chronic inflammation, has been demonstrated to be a critical molecular engine driving glomerular and tubulointerstitial damage [26].

### 3.1. High Glucose-Induced cGAS-STING Activation as the Initiating Trigger

In DN, persistent hyperglycemia is the initiating factor of all pathological changes. Exposure to elevated glucose levels triggers mitochondrial impairment, resulting in impaired function of the electron transport chain, abnormal elevation of membrane potential, and reduced antioxidant capacity, which results in excessive production of ROS. This subsequently causes oxidative damage and fragmentation of mtDNA [23]. When mitochondrial membrane permeability increases, damaged mtDNA leaks into the cytoplasm, becoming typical damage-associated molecular patterns (DAMPs) [63,64]. In the cytoplasm, cGAS acts as a DNA receptor. Following detection of free mtDNA, it undergoes activation, leading to cGAMP synthesis as a second messenger. The latter binds to and activates STING. Upon activation, STING subsequently moves to the Golgi apparatus and there recruits TBK1 along with I kappa B kinase (IKK) kinases. This ultimately activates the downstream transcription factors NF-κB and IRF3, initiating the expression of type I IFN and various pro-inflammatory factors [15,65]. Multiple studies have demonstrated that the expression and activity of STING are markedly increased in DN models, and are positively correlated with disease severity and progression [66,67]. This process constitutes the core signaling axis of “metabolic stress → DNA release → innate immune activation” in DN, providing sustained driving signals for subsequent inflammation, fibrosis, and cell death.

### 3.2. STING-Driven Glomerular Inflammation and Tissue Injury

Inflammation is a core pathological feature that emerges in the early stage of DN, characterized by monocyte/macrophage infiltration in the glomeruli, endothelial cell activation, and destruction of podocyte architecture. In this process, the STING pathway appears to act as an important amplifier of local immune responses through multidimensional mechanisms. On one hand, upon activation of STING, the NF-κB signaling cascade is engaged, leading to the release of numerous pro-inflammatory factors, including TNF-α, IL-1β, IL-6, and monocyte chemoattractant protein-1 (MCP-1). These factors not only directly damage glomerular intrinsic cells but also recruit peripheral immune cells for infiltration, thereby forming a persistent inflammatory microenvironment [26]. On the other hand, STING can activate the NLRP3 inflammasome through ROS-dependent or non-dependent mechanisms, promoting caspase-1 activation, thereby cleaving precursor IL-1β and IL-18 into mature forms, initiating an intense inflammatory response [57,68]. Ultimately, NLRP3 inflammasomes induce inflammation and pyroptosis in renal tubular cells [69]. Of particular significance, STING can also be activated in glomerular cells by a high-glucose environment, leading to disordered cytoskeletal remodeling and downregulation of key slit diaphragm proteins. This disrupts the integrity of the glomerular filtration barrier, ultimately resulting in proteinuria, the core clinical manifestation of DN [27]. Human tissue analyses have found that STING overexpression in the glomerular region of DN samples, supporting the activation of this pathway in human disease, while in vitro experiments using human-derived podocytes and animal studies further indicate that STING activation can contribute causally to albuminuria and podocyte loss [26]. Therefore, the strongest causal evidence currently comes from in vitro and animal models, whereas human data mainly support association and pathway activation.

Therapeutic interventions targeting STING, such as the use of inhibitors or natural compounds, including polydatin and sinomenine, have been reported to suppress the production of inflammatory cytokines and improve renal pathology in diabetic experimental models, underscoring the role of STING-mediated inflammation in DN [70,71]. For instance, the inhibition of proprotein convertase subtilisin/kexin type 9 (PCSK9) has been demonstrated to decrease mtDNA lesions and block the activation of the cGAS-STING axis, thereby alleviating inflammatory responses and improving renal function in diabetic mouse models [67]. These findings suggest that persistently activated STING signaling in diabetic kidneys may serves as a mechanistic connection bridging hyperglycemia-induced cellular stress and the inflammatory processes driving the progression of DN, while clinical efficacy in humans remains to be established.

### 3.3. STING-Mediated Renal Interstitial Fibrosis and Tissue Remodeling

**Figure 2:**
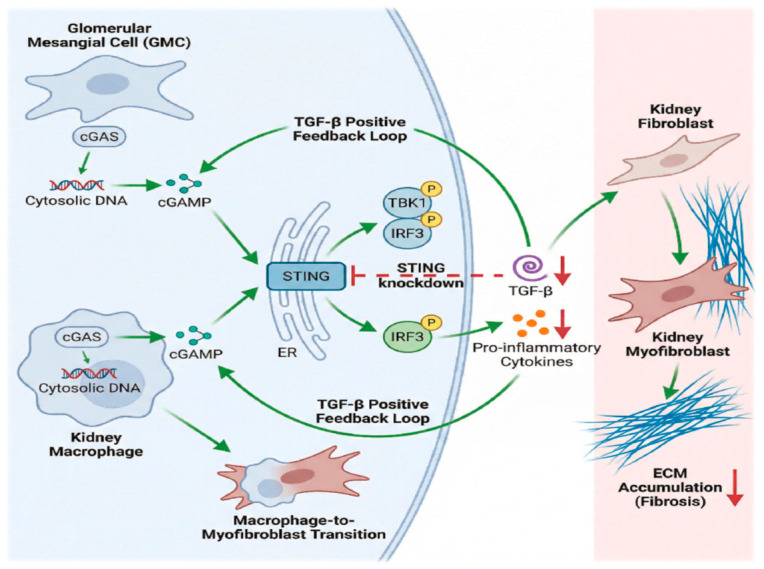
STING signaling promotes renal fibrosis through TGF-β positive feedback loop. STING signaling, stimulated by cytosolic DNA, drives the expression of TGF-β and pro-inflammatory cytokines. TGF-β promotes macrophage-to-myofibroblast transition and activates resident fibroblasts, thereby promoting ECM accumulation and renal fibrosis. This creates a TGF-β positive feedback loop that exacerbates fibrotic progression. Conversely, STING knockdown is proposed to interrupts this loop, reducing TGF-β levels and mitigating fibrosis. Abbreviations are specified in Abbreviations Section.

If chronic inflammation is not adequately controlled, renal tissue may progress toward fibrosis, with the STING pathway serving as an important contributor to this inflammation-fibrosis transition. Renal fibrosis is a pathological feature of progressive DN, primarily characterized by excessive deposition of ECM and loss of normal renal structure [72]. The STING signaling pathway contribute to fibrosis formation by modulating inflammatory and fibrotic signaling cascades. When renal cells activate STING, it promotes the production of pro-inflammatory cytokines and growth factors such as TGF-β, a key factor in fibroblast activation and ECM production [11,71]. Investigations have revealed that the cGAS-STING axis can induce phosphorylation of downstream effectors such as TBK1 and IRF3, thereby activating fibroblasts and upregulating fibrotic mediators [73,74]. Conversely, downregulation of STING reduces pro-inflammatory and fibrotic factors in high-glucose-stimulated glomerular mesangial cells, whereas excessive STING expression promotes inflammatory fibrosis. The role of STING signaling in promoting renal fibrosis via a TGF-β positive feedback loop is presented in Figure 2. Notably, the STING-mediated signaling pathway can also promote macrophage-to-myofibroblast transition and activate resident fibroblasts, thereby exacerbating fibrotic progression [74]. This mechanism has mainly been demonstrated in preclinical renal fibrosis models: profibrotic factors such as TGF-β1 stimulate macrophages, leading to mitochondrial DNA leakage, cGAS ac-tivation, cGAMP production, and STING activation; activated STING then recruits and phosphorylates TBK1, activating transcription factors, including IRF3 and HIF1α. This signaling cascade promotes acquisition of myofibroblast-like features, including α-smooth muscle actin and extracellular matrix protein expression [74]. The proposed interaction between the TGF-β signaling pathway and STING activation may form a positive feedback loop, although its detailed molecular regulation in human DN requires further study.

In chronic kidney disease models, therapeutic strategies that inhibit STING, such as small-molecule inhibitors or the traditional Chinese medicine preparation Shenbing Erhao Tang, have been demonstrated to reduce fibrosis and maintain mitochondrial function [75]. In a recent DN-focused study, STING deficiency alleviated renal fibrosis by inhibiting ID1-mediated epithelial–mesenchymal transition (EMT), suggesting that the STING-ID1 axis may represent a potential therapeutic target [76]. Mechanistically, STING was significantly upregulated in renal tubular cells of DN patients and negatively correlated with kidney function. Under glucolipotoxic conditions, STING activation in renal tubular epithelial cells upregulated ID1, a transcription factor that drives EMT by suppressing epithelial markers while inducing mesenchymal markers like α-SMA and fibronectin. Functional rescue experiments supported ID1 as a downstream effector of STING, and genetic or pharmacological inhibition of STING ameliorated renal fibrosis in DN mouse models [76]. However, because this mechanism is supported mainly by one recent clinical-sample-associated and preclinical study, independent validation and prospective human data are needed before it can be considered an established therapeutic axis in DN.

While the above findings collectively support a pathogenic role of STING in DN-associated renal fibrosis, several mechanisms remain emerging findings that require further validation: the STING-ID1-EMT axis has been reported in a recent study but awaits independent replication; the precise molecular details of the STING-TGF-β positive feedback loop—particularly how TGF-β signaling reciprocally regulates STING expression or activity—remain to be fully elucidated; the therapeutic efficacy of Shenbing Erhao Tang and the combination strategy of targeting both STING and ID1 are currently supported by preclinical evidence only, with no human data available to date. These emerging areas represent important directions for future investigation.

### 3.4. STING-Induced Renal Cells: Apoptosis, Necroptosis, and Pyroptosis

**Figure 3:**
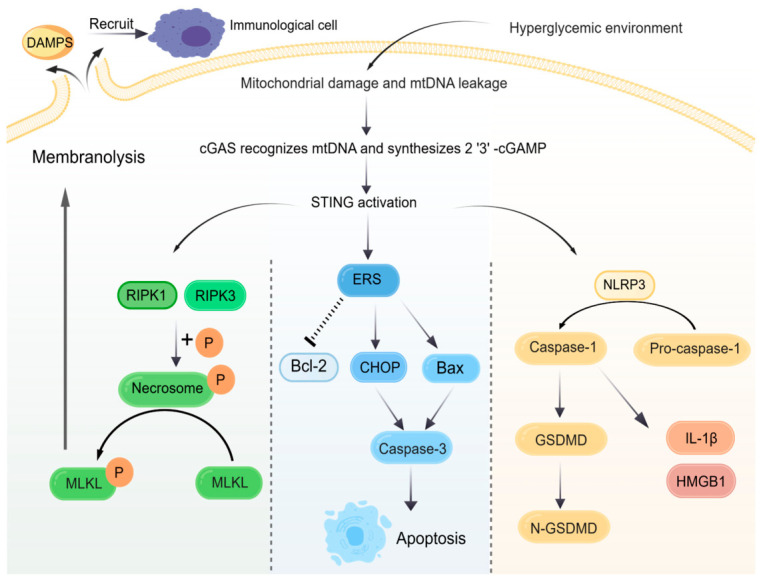
Three different types of programmed cell death triggered by STING activation. ERS mediates apoptosis through upregulation of CHOP and Bax, suppression of Bcl-2, and activation of caspase-3. RIPK-dependent necroptosis is induced, characterized by cellular swelling, plasma membrane rupture, and liberation of damage-associated molecular patterns. NLRP3 inflammasome activation leads to pyroptosis, involving GSDMD cleavage and IL-1β secretion. These pathways collectively contribute to nephron loss and renal fibrosis in DN. Abbreviations are specified in Abbreviations Section.

In DN, the activation of the STING signaling pathway not only triggers inflammatory responses and fibrosis but also directly leads to renal cell death by inducing apoptosis and necrosis, especially within kidney tubular epithelial cells and interstitial cells [77]. STING activation promotes the expression of interferon-regulated factors and pro-apoptotic mediators, thereby inducing programmed cell death and ultimately impairing renal function. The three types of programmed cell death triggered by STING signaling in DN are presented in Figure 3, and their cell-type specificity is summarized in Table 1.

First, STING activation can induce ERS, enhance the expression of pro-apoptotic factors, including C/EBP homologous protein (CHOP) and Bcl-2-associated X protein (Bax), while at the same time suppressing the anti-apoptotic regulator Bcl-2, which in turn triggers the caspase-3-mediated apoptotic cascade in renal tubular epithelial cells and podocytes [78]. Additionally, the interaction between STING activation and mitochondrial dysfunction exacerbates oxidative stress, disrupts cellular homeostasis, and accelerates the progression of renal injury across multiple renal cell types [79,80].

Second, when the STING signal is over-or persistently activated, it can trigger receptor interaction protein kinase (RIPK)-mediated necrotic apoptosis primarily in tubular epithelial cells, marked by cellular swelling, membrane rupture, and the efflux of abundant DAMPs, further amplifying local inflammation [81].

Third, STING can also promote pyroptosis by activating the NLRP3 inflammasome, which is a pore-forming GSDMD (Gasdermin D)-mediated cell lysis in both renal tubular epithelial cells and podocytes, accompanied by massive release of high mobility group protein 1 (HMGB1) and IL-1β, thereby establishing a vicious cycle of “inflammation, cell death, and re-inflammation” [82].

Notably, persistent activation of STING can impair mitochondrial biosynthesis and function, reduce ATP production, exacerbate cellular energy crisis, and thus predispose cells to irreversible death programs in various renal cell types [83]. El-Deeb et al. analyzed blood samples from 45 patients with different stages of DN and found that STING levels were significantly positively correlated with pyroptosis-related markers, such as NLRP3, caspase-1, and IL-1β, providing direct evidence for the involvement of STING in inflammatory cell death in human DN [57]. Persistent apoptosis and necrosis of renal cells not only reduce the number of nephrons but also create a fibrotic environment, thereby promoting the progression of DN [84].

Collectively, the pathogenic roles of STING in DN inflammation, fibrosis, and cell death suggest multiple therapeutic opportunities. These include small-molecule STING inhibitors like C-176 and H-151; natural compounds like paclitaxel and polydatin; PCSK9 inhibition, and the traditional Chinese medicine Shenbing Erhao Tang. Emerging strategies such as targeting the STING-ID1 axis also show promise. A detailed discussion of these therapeutic approaches, including their mechanisms, preclinical evidence, and clinical translation prospects, is provided in the following section.

## 4. STING-Targeted Therapeutic Strategies for DN

### 4.1. Small-Molecule STING Inhibitors and Pathway Modulators

#### 4.1.1. Direct STING Inhibitors

The development of small-molecule inhibitors targeting the STING pathway has garnered significant attention, owing to the involvement of STING in DN-associated inflammatory responses. To date, multiple STING inhibitors with varying targeting specificities have been reported, designed to interfere with different stages of STING activation. Studies have demonstrated that small-molecule inhibitors such as C-170 and C-176 exert their effects by directly acting on STING itself. Both agents block STING palmitoylation through covalent modification of the Cys91 site, thereby blocking STING activation and associated downstream cascades. Among them, C-170 can act on both human and murine STING, whereas C-176 is a murine-specific STING inhibitor. These compounds effectively suppress STING-mediated inflammatory responses and reduce the production of inflammatory cytokines in preclinical inflammatory disease models, providing mechanistic support for potential intervention in DN but not yet DN-specific clinical evidence [42,43].

Similarly, H-151 blocks STING activation and inhibits subsequent IRF3 phosphorylation by covalently binding to key cysteine residues of STING, significantly reducing pro-inflammatory cytokine expression. In contrast, polydactylin has been reported in DN models to reduce STING protein expression and inhibit downstream NF-κB nuclear translocation and TBK1 phosphorylation, ultimately suppressing pro-inflammatory and pro-fibrotic mediators [71]. The precision with which direct inhibitors engage their molecular targets is critically important, given that STING activation promotes the phosphorylation of TBK1 and IRF3. This molecular event subsequently drives the expression of type I interferons (IFNs) and multiple pro-inflammatory cytokines, including IL-1β, TNF-α, and MCP-1, all of which may contribute to kidney inflammation and fibrosis in DN [85]. By directly targeting STING, these inhibitory agents may interrupt the downstream inflammatory signaling cascade, although DN-specific efficacy varies among compounds and remains largely preclinical.

#### 4.1.2. Indirect cGAS-STING Signaling Axis Modulators

Furthermore, certain natural products, such as sinomenine, have been reported to indirectly suppress the cGAS-STING signaling axis. This suppression is achieved through the downregulation of essential inflammatory mediators by inhibiting the p-TBK1, p-IRF3, and NF-κB signaling pathways, thereby mitigating renal inflammatory damage in diabetic mouse models [70]. These agents should therefore be interpreted as indirect or multi-target modulators rather than selective STING inhibitors.

**Figure 4:**
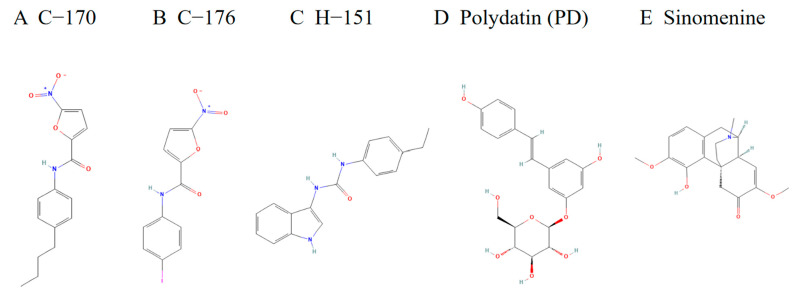
Chemical structures of STING-related inhibitors and agents summarized in Table 2.

Collectively, the therapeutic value of STING-targeted inhibitors resides not solely in their capacity to reduce inflammation, but also in their ability to limit aberrant STING activation resulting from mtDNA leakage, which represents a crucial upstream event in DN pathogenesis [67,86]. Current evidence supports small-molecule STING-targeting agents as promising experimental therapeutic options for modulating innate immune responses and alleviating inflammatory renal injury in DN. However, compounds with direct DN-specific evidence should be distinguished from agents whose rationale is extrapolated from other inflammatory disease contexts. The chemical structures of the five STING-related inhibitors or agents summarized in Table 2 are shown in Figure 4.

As summarized in Table 2, the structural features of these agents are closely related to their mechanisms of STING pathway modulation [19,87]. C-170, C-176, and H-151 are small-molecule covalent STING inhibitors whose electrophilic structural motifs enable interaction with key cysteine residues of STING, particularly Cys91, thereby preventing activation-induced palmitoylation [87,88]. Because STING palmitoylation is required for Golgi-associated clustering and downstream TBK1/IRF3 activation, these structural properties contribute to reduced type I interferon production and NF-κB-mediated inflammatory cytokine release [19,88]. In DN, however, the therapeutic implications of these direct covalent inhibitors are mainly inferred from STING biology and non-DN inflammatory models, and therefore require DN-specific validation [30,67].

In contrast, polydatin (PD) and sinomenine are better interpreted as indirect or multi-target modulators. Their effects on STING-associated physiological events may involve broader regulation of oxidative stress, mitochondrial dysfunction, and inflammatory signaling rather than a single defined cysteine-targeting mechanism [70,71]. This distinction is important because direct covalent STING inhibitors may offer stronger pathway specificity, whereas indirect modulators may provide broader anti-inflammatory effects but require careful validation of target specificity, dose–response relationships, and disease-stage applicability [87]. Thus, among the agents discussed, polydatin and sinomenine currently have more DN-model-related evidence, while C-170, C-176, and H-151 remain primarily mechanistic or extrapolated candidates for DN.

#### 4.1.3. Challenges in STING-Targeted Therapy

Despite the promising therapeutic potential of STING-targeted agents, several critical limitations must be acknowledged before their clinical application in DN.

One major concern is the specificity of current STING inhibitors. While compounds such as C-176 and H-151 have demonstrated potent inhibitory effects in preclinical models, their selectivity profiles remain incompletely characterized. Off-target degradation represents a significant risk, particularly when using CRBN-based E3 ligase recruiters with broad expression profiles [89]. Furthermore, C-176 is specific to murine STING due to species-specific differences in the palmitoylation pocket and cannot be directly translated to human applications. Indeed, well-characterized species selectivity has been documented, with DMXAA showing potent activity in mouse STING but only partial agonistic activity in human STING [90]. Of note, this 2024 study revealed that DMXAA is actually a partial STING agonist that interferes with agonistic STING activation in humans, rather than being completely inactive as previously thought. This species specificity necessitates the development of human-specific inhibitors, and while C-170 exhibits activity against both human and murine STING, further optimization is still required.

Safety considerations pose another substantial hurdle. The STING pathway plays a fundamental role in innate immune surveillance against both pathogens and malignant cells. Chronic or systemic inhibition of STING could therefore increase susceptibility to infections or impair DNA damage responses, potentially compromising cancer immunosurveillance. Preclinical studies have shown that sustained STING suppression may disrupt immune homeostasis and increase vulnerability to viral infections, as STING is essential for detecting cytosolic DNA from pathogens [17]. Importantly, the potential for cytokine release syndrome has been observed in STING agonist trials. In the first-in-human study of the STING agonist SYNB1891, five cytokine release syndrome events occurred, including one dose-limiting toxicity at the highest dose [91]. Similarly, the STING agonist MIW815 (ADU-S100) combined with the PD-1 inhibitor spartalizumab was associated with adverse events, including pyrexia (22%), injection site pain (20%), and diarrhea (11%) [92]. These observations underscore that STING pathway modulation—whether activation or inhibition—must be carefully balanced to avoid unintended inflammatory or immunosuppressive consequences.

The translational challenges for STING-targeted therapies are substantial. To date, no STING inhibitor has entered clinical trials, with most candidates still in preclinical stages of development for DN and other inflammatory diseases. Clinical efforts have instead focused on STING agonists for cancer immunotherapy, yet even these have encountered significant obstacles. The STING agonist ADU-S100 (MIW815) as monotherapy showed limited antitumor activity; in a Phase Ib study combining it with the PD-1 inhibitor spartalizumab, the overall response rate was only 10.4% [92]. Similarly, the STING agonist IMSA101 in a Phase I first-in-human study showed minimal antitumor activity, with no complete or partial responses in the monotherapy arm and an overall response rate of only 5.6% in the combination arm [93]. These disappointing results have been attributed to poor pharmacokinetic stability, suboptimal cellular uptake, and the inability to achieve sustained STING activation within the target tissue without systemic toxicity [94]. Moreover, the successful application of STING inhibitors in DN would require cell-type-specific delivery strategies, given the dual role of STING in different renal cell populations—protective in some contexts but pathogenic in others [95]. The need to maintain physiological STING functions while blocking pathological activation remains an unresolved challenge.

### 4.2. Gene-Based Interventions in STING Regulation

Gene intervention technologies have become powerful experimental tools for regulating STING expression and activity in DN models, including RNAi and gene editing technologies. Persistent hyperglycemia in DN triggers mitochondrial DNA leakage and activates the cGAS-STING pathway, driving three core pathological events: chronic inflammation, renal fibrosis, and death of renal tubular epithelial cells. Therefore, targeting STING directly using gene intervention strategies may help clarify and potentially interrupt this pathogenic cascade at its source, although therapeutic application remains at an early preclinical stage. RNAi reversibly downregulates gene expression by introducing small RNA molecules such as siRNA or shRNA to specifically degrade target mRNA or inhibit its translation at the post-transcriptional level, without altering the genomic DNA sequence [96]. In contrast, gene editing technologies represented by CRISPR-Cas9 directly perform precise cleavage and modification of genomic DNA, enabling permanent gene knockout, insertion, or point mutation, thereby generating stable genetic alterations in the genome [97,98].

RNAi technology is primarily employed to regulate the expression level of the STING gene. By utilizing siRNA and shRNA technologies targeting STING mRNA, researchers have successfully inhibited the expression of this protein both in vitro and in vivo, thereby alleviating the pathological alterations associated with DN. For instance, using an HK-2 renal tubular epithelial cell model cultured under high-glucose conditions in vitro, siRNA-mediated STING silencing significantly reduced the expression of pro-inflammatory cytokines such as TNF-α and IL-6, while alleviating mtDNA damage and intracellular mitochondrial stress responses. This indicates that STING inhibition effectively mitigates cellular inflammation and oxidative stress induced by high-glucose conditions [99]. This further corroborates how STING activation drives inflammatory responses through NF-κB and exacerbates the pathological process of glomerular injury. Furthermore, relevant studies have also found that RNAi technology is not limited to simply inhibiting STING, but can also regulate other key molecules in the cGAS-STING pathway, thereby further optimizing the modulation of inflammatory cascades [100]. Specifically, by blocking the cGAS-STING signaling pathway, it inhibits the initiation of downstream functional effectors such as TBK1 and NF-κB, these factors are the “drives”of inflammatory responses and the progression of DN.

In addition to RNAi technology, gene-editing techniques such as CRISPR-Cas9 have provided novel approaches for more precise and sustained modulation of STING pathway components. Although direct application studies of the STING gene in DN models remain insufficient, the potential of this technology for gene sequence knockout or modification has opened new directions for future therapeutic strategies to suppress chronic inflammation in diabetic kidneys [101]. Gene-editing technologies hold promise for mitigating the inflammatory progression and renal function decline in DN by repairing or knocking out aberrant STING; gene editing technology can also correct abnormal STING activation associated with mitochondrial dysfunction and mtDNA leakage, which are key mechanisms underlying the progression of DN [86]. For example, given that STING drives renal fibrosis in DN through multiple mechanisms, such as promoting macrophage-to-myofibroblast transition (MMT) via TBK1 activation, and inducing epithelial–mesenchymal transition (EMT) of renal tubular epithelial cells by upregulating ID1, which in turn suppresses E-cadherin and induces α-SMA and fibronectin expression, CRISPR-Cas9-mediated permanent knockout of STING holds promise for interrupting these pro-fibrotic signaling pathways at the genomic level. For DN, human pluripotent stem cells (hPSCs) and induced pluripotent stem cells (iPSCs) combined with CRISPR technology offer a platform to model STING-related renal pathology and screen gene repair strategies targeting the STING pathway, particularly suitable for personalized therapy and drug discovery [102,103]. More importantly, gene editing can also be utilized to optimize cell therapy vectors, thereby enhancing the safety and efficacy of treatments [104].

Despite its potential, RNAi often faces off-target effects and limited duration of efficacy. Off-target effects arise mainly because the seed region of siRNA may partially pair with non-targeted mRNAs, leading to unintended gene silencing [105]. In the context of DN, where renal cells are chronically exposed to hyperglycemia and oxidative stress, this off-target risk is amplified—inadvertent silencing of genes essential for tubular epithelial cell survival or podocyte cytoskeletal maintenance may paradoxically exacerbate renal injury. Delivery also poses challenges. Naked RNA molecules are susceptible to nuclease degradation and the uptake efficiency of delivery carriers varies markedly across different renal cell types [106]. Given that DN pathology involves multiple cell types—including tubular epithelial cells, podocytes, and glomerular mesangial cells—insufficient delivery efficiency directly compromises therapeutic outcomes.

CRISPR-Cas9 faces similar challenges in off-target effects and delivery. Off-target risk arises from Cas9 cutting similar genomic sequences. In DN, the diseased kidney microenvironment further influences off-target profiles and delivery efficiency [107]. For delivery, SpCas9 (~4.2 kb) exceeds AAV capacity (~4.7 kb), requiring alternatives. The smaller SaCas9 (~3.2 kb) fits into a single AAV with fewer off-target effects [108]. In DN, LNP-delivered CRISPR RNP reduces the pro-fibrotic factor TGFB1 by 67%, offering a strategy to block fibrotic progression [109]. Moreover, kidney-specific promoter-mediated base editing has achieved targeted gene correction in DN animal models without extra-renal off-target effects [110], laying a foundation for precision gene therapy in DN.

In summary, RNAi, with its rapid and flexible gene expression regulation capability, achieves target gene silencing within a short period, making it suitable for temporarily suppressing abnormal gene expression. However, RNAi often faces off-target effects and limited duration of efficacy. In contrast, gene editing technology enables permanent and precise modifications to the genome, providing potentially durable therapeutic effects, but delivery efficiency, safety, and off-target gene editing remain major barriers to clinical application [111,112]. The integration of RNAi with gene editing technology provides an experimental platform for elucidating the molecular mechanisms of STING in DN and for developing more targeted treatment concepts. At present, gene-based STING-targeted therapies should be regarded as a frontier research direction rather than an established therapeutic option for DN.

### 4.3. Combination Therapy Strategies and Preclinical Studies

In recent years, preclinical investigations have centered on the synergistic effects of combining sodium-dependent glucose transporters 2 (SGLT2) inhibitors with renin–angiotensin–aldosterone system (RAAS) inhibitors in animal models of DN, suggesting that combination therapy may provide more significant renal and cardiovascular benefits compared to monotherapy [113]. This suggests that combining STING inhibitors with existing treatments for DN may be a viable strategy to enhance therapeutic efficacy and reduce adverse effects. Although direct combination studies of STING inhibitors with RAAS or SGLT2 inhibitors are limited, their theoretical basis is supported by complementary mechanisms: RAAS inhibitors can reduce intraglomerular hypertension and fibrosis, SGLT2 inhibitors improve glycemic control and renal hemodynamics, while STING inhibitors specifically inhibit innate immune-mediated inflammation. Specifically, RAAS inhibitors can markedly decrease the production of pro-inflammatory mediators such as TNF-α and IL-1β by inhibiting the NF-κB pathway and synergizing with the IRF3/NF-κB signaling downstream of STING, thus mitigating kidney inflammation and fibrotic changes [114]. In contrast, SGLT2 inhibitors indirectly inhibit the triggering of the cGAS-STING axis by improving mitochondrial function and reducing mtDNA leakage, thereby blocking the inflammatory cascade [71]. Safety evaluations in animal models demonstrated that STING inhibition does not exacerbate systemic immunosuppression, which is critical for combination therapy regimens [65]. Moreover, some glucagon-like peptide-1 (GLP-1) agonists demonstrate vascular protective properties through suppression of the STING signaling axis in models of diabetic kidney disease. Alternatively, they may serve a complementary function in DN therapy by mitigating endothelial inflammatory responses.

Importantly, the rationale for combining STING modulation with RAAS inhibitors, SGLT2 inhibitors, or GLP-1 receptor agonists is currently based mainly on complementary mechanisms and preclinical observations. Direct DN studies testing these combinations are still limited, so this strategy should be considered hypothesis-generating rather than clinically established. These preclinical study results indicate that integrating STING pathway modulation into existing DN treatment regimens may hold therapeutic potential. Future clinical trials are required to evaluate the efficacy, safety, patient-selection criteria, and optimal dosing regimens of such combination therapies, with the aim of controlling metabolic, hemodynamic, and inflammation-related factors in the progression of DN.

### 4.4. Biomarkers for Monitoring STING Activation and Patient Selection

The successful clinical translation of STING-targeted therapies in DN relies on reliable methods to identify patients most likely to benefit. Non-invasive assessment of STING pathway activation through blood or urine testing offers a feasible strategy for patient stratification and personalized treatment.

#### 4.4.1. Blood-Based Biomarkers

El-Deeb and colleagues examined blood samples from DN patients at different stages of albuminuria and found that STING mRNA and protein levels were significantly elevated, with the highest values observed in the macroalbuminuric group (*p* < 0.001). Serum STING levels also showed strong positive correlations with pyroptosis markers NLRP3, caspase-1, and IL-1β. In addition to STING, the same study revealed that AIM2 (another cytosolic DNA sensor) and IRF3 (a direct downstream effector of STING) were similarly elevated in DN patients, suggesting that measuring a panel of cGAS-STING pathway components could comprehensively assess the inflammatory cascade [15].

#### 4.4.2. Urine-Based Biomarkers

Mitochondrial DNA (mtDNA) has emerged as a promising urinary biomarker reflecting STING activation. Mitrofanova and co-workers demonstrated that urinary mtDNA levels are elevated in diabetic db/db mice and correlate with both STING activation and podocyte injury. Free mtDNA was detectable in urine, and mtDNA administration induced STING phosphorylation and albuminuria, while STING-deficient mice remained protected from injury. Given that the cGAS-STING pathway is triggered by mtDNA leakage under cellular stress, urinary mtDNA detection has been proposed as a non-invasive surrogate marker for intrarenal STING signaling. Jin and colleagues further noted that abnormal mtDNA copy number is associated with kidney disease progression, highlighting its diagnostic and prognostic potential [65]. In addition, classical kidney injury markers such as KIM-1 and NGAL can be detected in urine to complement STING-related biomarkers [115]. EGR1, an upstream regulator of STING that is upregulated in DN podocytes, may serve as an early warning indicator [116].

#### 4.4.3. Clinical Prospects and Challenges

Using blood (for STING, AIM2, IRF3, and inflammatory markers) and urine (for mtDNA and kidney injury markers) to monitor STING activation enables non-invasive pathway assessment, facilitates patient stratification in clinical trials, and guides personalized therapy. For example, patients with elevated serum STING or urinary mtDNA levels could be prioritized for enrollment in STING inhibitor trials. Before these biomarkers can be integrated into routine clinical practice, several obstacles must be overcome. Standardized protocols for measuring STING protein levels, mtDNA, and other related markers require validation across multiple laboratories. Large-scale prospective studies are needed to establish appropriate cut-off values, determine the sensitivity and specificity of these assays across diverse patient populations, and assess whether biomarker-guided patient selection translates into improved treatment outcomes. Furthermore, the dynamic changes in these biomarkers during disease progression and in response to therapeutic intervention warrant further investigation.

In summary, blood-based detection of STING, AIM2, and IRF3, together with urine-based detection of mtDNA and kidney injury markers, represent promising non-invasive strategies for evaluating STING pathway activation and guiding targeted therapy in DN patients.

## 5. Conclusions

Diabetic nephropathy remains a leading cause of end-stage renal disease, and current therapies often fail to halt disease progression in a substantial proportion of patients. This review summarizes evidence suggesting the STING signaling pathway may function as a central molecular hub driving the interconnected “inflammation–fibrosis–cell death” cascade in DN. Specifically, STING activation promotes the release of pro-inflammatory cytokines, contributes to the transition from inflammation to fibrosis via TGF-β-related signaling and macrophage-to-myofibroblast transition, and triggers multiple programmed cell death pathways, including apoptosis, necroptosis, and pyroptosis. The strength of evidence differs across these mechanisms, with causal support strongest in cell and animal studies and more limited supportive evidence available from human DN samples.

Based on the evidence discussed in this review, several therapeutic strategies targeting STING show promise, and future efforts should move beyond single-target inhibition toward a more integrated approach. Small-molecule STING inhibitors have demonstrated efficacy in preclinical models by blocking STING palmitoylation or directly binding to STING, while indirect modulators suppress the cGAS-STING axis via downstream pathways. Gene intervention technologies, including RNAi and CRISPR-Cas9, enable STING silencing or pathway modulation at the genetic level, but their application in DN remains experimental. Looking beyond these direct targeting strategies, future directions should focus on three areas. First, developing cell-type or tissue-specific STING modulators, or utilizing smart nanocarriers for localized delivery, could balance therapeutic efficacy with systemic immune homeostasis. Second, integrating single-cell multi-omics, spatial transcriptomics and artificial intelligence technologies may help profile STING-related gene expression across different cell types like podocytes, tubular epithelial cells, and macrophages in DN tissues, thereby supporting STING-based molecular classification and patient stratification. Third, combining STING inhibition with existing therapies such as SGLT2 inhibitors or RAAS blockers may offer synergistic benefits by targeting multiple pathological axes simultaneously, though direct experimental evidence remains limited. In the longer term, gene editing or RNA interference technologies may offer possibilities for reshaping renal immune homeostasis after substantial validation of delivery, safety, and specificity.

However, several challenges must be acknowledged. For small-molecule STING inhibitors, specificity issues include species specificity (C-176 is murine-specific) and potential off-target effects. Safety concerns include the risk of immunosuppression as well as cytokine release syndrome observed in STING agonist trials. Clinical translation is hampered by the lack of human-specific inhibitors, delivery hurdles, and the dual role of STING in different renal cell populations. For gene intervention technologies, RNAi faces off-target effects and limited duration of efficacy, while CRISPR-Cas9 faces low delivery efficiency, Cas9 immunogenicity, and potential off-target editing risks. Moreover, cell-type-specific delivery in the kidney remains a technical hurdle for both approaches. Furthermore, the STING-ID1-EMT axis and other emerging mechanisms require further validation.

In summary, in the face of the severe global health challenge of DN, further investigation of STING-mediated inflammation, fibrosis, and cell-death signaling may help clarify disease mechanisms and identify new therapeutic opportunities. At the same time, the translation of STING-targeted strategies from experimental models to human DN will require rigorous validation of pathway specificity, patient selection, long-term safety, and added benefit over current standard therapies.

## Figures and Tables

**Table 1:** Cell-type specificity of STING-mediated cell death pathways in DN.

Cell Death Pathway	Key Mediators	Affected Cell Types in DN
Apoptosis	Caspase-3, CHOP, Bax	Tubular epithelial cells, Podocytes
Pyroptosis	NLRP3, Caspase-1, GSDMD	Tubular epithelial cells, Podocytes
Necroptosis	RIPK1/3, MLKL	Tubular epithelial cells

**Table 2:** STING inhibitors and their mechanisms of action.

Inhibitor	Target	Molecular Mechanism	Specificity/Applicable Model	Therapeutic Effects in DN or Inflammatory Models
C-170	Cys91 site of human and murine STING	Block palmitoylation, inhibit STING activation	human and murine	Inhibit type I IFN and pro-inflammatory factor production, alleviating inflammatory responses
C-176	Cys91 site of murine STING	Inhibit STING palmitoylation and activation	murine	Improved systemic inflammation and lowered levels of IL-1β, TNF-α, etc.
H-151	STING key cysteine residue	Block STING activation and inhibit IRF3 phosphorylation	human and murine, commonly used in human cells	Reduces the expression of type I IFN and pro-inflammatory cytokines
PD	direct binding to STING	Reduce STING expression thereby inhibiting TBK1 phosphorylation and NF-κB nuclear translocation	no species-specificity has been established	Inhibit pro-inflammatory and fibrotic factor production
Sinomenine	indirect inhibition of the cGAS-STING pathway	Downregulate p-TBK1, p-IRF3, and NF-κB activity	non-direct STING inhibitors	Reduce renal injury and decrease the expression of inflammatory mediators

## Data Availability

Not applicable.

## Abbreviations

Bax	Bcl-2-associated X protein
Caspase-1	Cysteine-aspartic acid protease 1
CDNs	Cyclic dinucleotides
cGAMP	Cyclic guanosine monophosphate-adenosine monophosphate
cGAS	Cyclic guanosine monophosphate synthase
CHOP	C/EBP homologous protein
CTT	C-terminal tail
DAMPs	Damage-associated molecular patterns
DN	Diabetic nephropathy
dsDNA	Double-stranded DNA
ECM	Extracellular matrix
EMT	Epithelial–mesenchymal transition
ER	Endoplasmic reticulum
ERS	Endoplasmic reticulum stress
ESRD	End-stage renal disease
EVs	Extracellular vesicles
FASN	Fatty acid synthetase
GFR	Glomerular filtration rate
GLP-1	Glucagon-like peptide-1
GMCs	Glomerular mesangial cells
GSDMD	Gasdermin D
HG	High glucose
HMGB1	High mobility group protein 1
hPSCs	Human pluripotent stem cells
IDI1	Isopentenyl diphosphate isomerase 1
IFN	Interferon
IKK	I kappa B kinase
IL	Interleukin
iPSCs	Induced pluripotent stem cells
IRF	Interferon regulatory factor
LBD	Ligand-binding domain
MCP-1	Monocyte Chemoattractant Protein-1
mtDNA	Mitochondrial DNA
NF-κB	Nuclear factor kappa-B
NLRP3	NOD-like receptor thermal protein domain associated protein 3
PCSK9	Protease-kexin 9
PPP6C	Protein phosphatase 6
RAAS	Renin–angiotensin–aldosterone system
RIPK	Receptor interaction protein kinase
RNAi	RNA interference
ROS	Reactive oxygen species
SGLT2	Sodium-dependent glucose transporters 2
STING	Stimulator of interferon genes
TBK1	TANK-binding kinase 1
TBK1	TANK binding kinase 1
TGF-β	Transforming growth factor-β
TNF-α	Tumor necrosis factor-α
TRIM	Tripartite motif containing
